# Reshaping neonatal intensive care units (NICUs) to avoid the spread of severe acute respiratory coronavirus virus 2 (SARS-CoV-2) to high-risk infants

**DOI:** 10.1017/ice.2020.310

**Published:** 2020-06-24

**Authors:** Domenico Umberto De Rose, Cinzia Auriti, Francesca Landolfo, Irma Capolupo, Guglielmo Salvatori, Stefania Ranno, Carlo Concato, Annabella Braguglia, Pietro Bagolan, Andrea Dotta

**Affiliations:** 1 Neonatal Intensive Care Unit, Department of Medical and Surgical Neonatology, “Bambino Gesù” Children’s Hospital IRCCS, Rome, Italy; 2 Virology Unit, “Bambino Gesù” Children’s Hospital IRCCS, Rome, Italy; 3 Neonatal Sub-Intensive Care Unit, Department of Medical and Surgical Neonatology, “Bambino Gesù” Children’s Hospital IRCCS, Rome, Italy; 4 Newborn Surgery Unit, Department of Medical and Surgical Neonatology, “Bambino Gesù” Children’s Hospital IRCCS, Rome, Italy

*To the Editor—*Italy has been hard hit by coronavirus disease 2019 (COVID-19), mostly in the northern regions,^1^ while the Lazio region was partially spared. Although no evidence thus far has shown vertical transmission,^2,3^ neonatologists are concerned about preventing the spread of infection-related disease (COVID-19) in infants and healthcare workers (HCWs) of neonatal intensive care units (NICUs).^4^ Here, we report our NICU’s reorganization during the COVID-19 pandemic.

## Methods

This observational study focused on infants admitted to our tertiary-care–level NICU during the COVID-19 outbreak in Italy from March 15 to May 15, 2020. Severe acute respiratory coronavirus virus 2 (SARS-CoV-2) was ruled out by nasopharyngeal swab in all infants before NICU admission. Detection of SARS-CoV-2 was performed using the Allplex 2019-nCoV assay (Seegene, Seoul, South Korea) as previously described.^5^

Nasopharyngeal swabbing was performed at referring center in stable neonates. SARS-CoV-2–positive neonates were managed in a dedicated pediatric COVID-19 unit. If the swab test was negative, the newborn was referred to our non–COVID-19 NICU as usual by neonatal emergency transport service (NETS) staff. For untested babies, accurate telephone contacts between spoke and hub centers were crucial to assuring the safest pathway for admission. Newborns were managed by HCWs wearing surgical masks (when COVID-19 was not suspected and/or mothers were negative within 24 hours of life) or N95 masks (in case of suspected or confirmed SARS-CoV-2–positive newborns), gloves, eye protection, and gowns. Upon arrival at our hospital, newborns were isolated and temporarily managed by an NETS dedicated neonatologist and nurse in a separate pavilion. The staff were equipped with personal protective equipment until the negative result of the nasopharyngeal swab for SARS-CoV-2 arrived. Thereafter, the neonate could be hospitalized in our NICU in a room with other infants.

Infants admitted to the emergency department (ED) were tested for SARS-CoV-2 on arrival and remained there until the negative result was available prior to NICU admission.

In the NICU, disposable surgical masks were always required for HCWs; body temperature was measured every day for all HCWs. Parental visits are still restricted to 6 hours each day, and only 1 parent is allowed for each baby, after signing a self-certification regarding fever, symptoms, and contacts. All parents are required to wear masks, gloves, and disposable clothing. If they have fever or respiratory symptoms the visit to the baby is forbidden, as reported in Veneto.^6^ Kangaroo-mother care has always been performed in our NICU. Through the institutional database, we identified all subjects hospitalized during this period and retrospectively analyzed medical records. As nasopharyngeal swab was requested by the health directorate of our institution, and no approval from our institution’s ethical committee was required for this study.

## Results

During the study period, 101 newborns required NICU admission; of these, 71 newborns were referred from other institutions and 30 infants who were firstly discharged from other institutions and then required rehospitalization were admitted from the ED (Table 1).

**Table 1.** Age on Admission of Infants Hospitalized in Our NICU From March 15, 2020, to May 15, 2020

Patients	Age at NICU Admission, median d (IQR)
All infants (n=101)	12 (2–27)
All infants referred from other institutions (n=71)	5 (1–23)
All infants admitted from emergency department (n=30)	21 (16–41)

Note. NICU, neonatal intensive care unit; IQR, interquartile range.

No newborns referred from other institutions tested positive for SARS-CoV-2.

Among infants admitted from the ED, only 1 neonate (3.3% of infants admitted from the ED), who required hospitalization because of urinary tract infection, tested positive via nasopharyngeal swab test for SARS-CoV-2. Considering his stable conditions, the infant was hospitalized in the dedicated pediatric COVID-19 unit and not in NICU; a neonatologist gave requested advices for the appropriate management. No COVID-19 cases were registered in infants hospitalized in the NICU under the adopted procedures and avoiding any contact with COVID-19 cases. No HCWs acquired COVID-19.

## Discussion

One of the lessons learned from the COVID-19 pandemic in Italy is that hospital overcrowding was a serious mistake that led hospitals to become the first places to spread the infection.^1^ Although concern about COVID-19 severity is less in children,^7^ the amount of SARS-CoV-2 viral material that could be potentially harmful in neonates remains unknown, especially in preterm infants.^2^ Therefore, strict preventive measures should be adopted in all NICUs. The relative ease of horizontal transmission, even within a controlled NICU, has been highlighted.^8^

Our NICU is a referral care center for severely ill neonates, with a delivery room only for highly selected patients. Therefore, almost all babies are referred from Rome and Lazio, and other regions either as a planned or an emergency admission because of a variety of high-risk conditions: surgical emergencies, congenital heart defects, perinatal asphyxia, prematurity, genetic syndromes, infections, metabolic disorders, etc. NETS play a key role in minimizing the risk of contamination from one institution to another,^9^ which is especially important in an outborn NICU such as ours. However, none of the infants referred from other institutions tested positive for SARS-CoV-2 during the study period. On the contrary, infants admitted from the ED, including the sole neonate who screened positive for SARS-CoV-2, had a higher age at admission and thus they had potentially been more exposed to the community. Differences between the 2 groups were not calculated because of the small sample size and a sole neonate who tested positive. The main limitation of our study was the single center site in a medium-risk region, and 70% of these cases were registered in northern Italy. However, the procedures outlined here allowed us to ensure continuity of care in the NICU, with limited risks of infection for newborns and HCWs. Although no newborns screened positive among NICU patients in the higher-risk Veneto region,^6^ by testing all newborns before admission, we could have separated a positive neonate from other fragile infants, avoiding life-threatening risks for them and the incalculable costs of spreading the virus to the unit. The fight against the COVID-19 pandemic should not affect the quality of the assistance to high-risk infants. Therefore, we conclude that all newborns should be tested for SARS-CoV-2 before being admitted to NICUs, especially if admitted from an ED.
